# Applying SDT and JD-R: A Practical Guide to Systemic Wellness Reforms in Medical Education

**DOI:** 10.12688/mep.20886.1

**Published:** 2025-04-16

**Authors:** Adam Neufeld

**Affiliations:** 1 Department of Family Medicine, University of Calgary, Calgary, Alberta, Canada

**Keywords:** physician wellness, burnout prevention, Self-Determination Theory (SDT), Job Demands-Resources (JD-R) Theory, medical education reform, basic psychological needs

## Abstract

**Background:**

Physician wellness is a growing concern in medical education, with burnout rates among medical professionals continuing to rise. While there is increasing recognition that systemic reforms are essential to addressing this issue, there is a significant gap in theory-based frameworks to guide the implementation of such reforms. Without a shared theoretical language or guiding principles, efforts to reform medical education often lack coherence, making it difficult to evaluate their effectiveness or replicate successful strategies.

**Methods:**

This paper aims to fill this gap by applying Self-Determination Theory (SDT) and the Job Demands-Resources (JD-R) model to create a concrete, theory-informed framework for systemic reform in medical education. I present 12 actionable recommendations that focus on two key areas: (1) reducing hindrance job demands that frustrate basic psychological needs and contribute to burnout, and (2) increasing job resources that promote need satisfaction and well-being. These recommendations are designed to provide a clear and scalable approach for institutions to adopt.

**Results:**

The recommendations in this paper offer practical strategies for optimizing learning environments in medical education. The strategies focus on enhancing physician autonomy, competence, and relatedness by addressing both job demands that hinder well-being and resources that foster engagement and growth. By applying SDT and JD-R as guiding principles, these reforms ensure that systemic changes are measurable and sustainable over the long term.

**Conclusions:**

This paper provides a theory-guided framework for systemic reforms aimed at promoting physician wellness in medical education. By applying the SDT and JD-R theories, medical educators and institutions can implement targeted strategies to reduce burnout and foster a thriving work environment. The framework not only offers actionable recommendations but also a means of measuring the impact and effectiveness of these reforms, ensuring their long-term success in improving physician well-being.

## Introduction

Physician burnout is a growing crisis in medical education, with increasing reports of stress, emotional exhaustion, and disengagement among trainees and faculty ^1^. Despite widespread recognition that systemic factors—such as excessive workloads, hierarchical cultures, and rigid curricula—are key drivers of burnout ^2^, there remains a lack of consensus on how to effectively address these issues at a structural level. While systemic change is widely acknowledged as the next necessary step ^1^, and various articles have been published discussing system-based interventions to address the root causes of burnout ^3– 5^, there is an absence of clear, theory-informed frameworks to guide this transformation. As a result, efforts to reform medical education are often fragmented and lack guiding principles, making it difficult for institutions and leaders, such as healthcare chief wellness officers ^6^, to design interventions that are both reliable and scalable.

Self-Determination Theory (SDT) ^7^ and the Job Demands-Resources (JD-R) ^8^ theory provide valuable insights into the relationship between workplace design, motivation, and well-being. SDT highlights how the fulfillment of basic psychological needs—autonomy, competence, and relatedness—underpins well-being, while JD-R emphasizes the interaction between job demands and resources in shaping stress and burnout. However, while both theories have been extensively studied in organizational psychology ^9, 10^, their application to medical education has been limited, particularly in the context of systemic reforms. Moreover, while momentum is building toward addressing the systemic roots of burnout, there is still a lack of concrete, theory-guided approaches that can be directly implemented in medical training environments.

This paper offers a clear, actionable framework for systemic reform in medical education, grounded in SDT and JD-R, with 12 practical recommendations aimed at addressing the systemic drivers of burnout. These recommendations are organized into two main categories: (1) reducing hindrance job demands that frustrate basic psychological needs, and (2) increasing job resources that satisfy these needs. Designed to promote flourishing and sustainable performance (i.e.,“Physician well-being 2.0” ^1^), they provide a shared roadmap for medical educators to create more supportive environments. Blending these macro-theories offers a flexible, theory-informed approach that clarifies key concepts, shows how they interact, and highlights the role of individual and environmental factors in shaping human engagement, performance, and wellness. This innovative combination of SDT and JD-R provides the tools institutions need, without prescribing rigid implementation methods, ensuring that reforms are adaptable to each institution's unique context. Ultimately, the theory-based reforms offer a reliable, scalable way forward to promote physician wellness that is measurable, replicable, and impactful.

## Structural reforms: reducing demands that frustrate basic psychological needs

To effectively address the systemic causes of physician burnout, medical education must prioritize reforms that reduce job demands that frustrate basic psychological needs, as well as increase resources that satisfy those needs and promote well-being. The following recommendations are grounded in SDT and the JD-R theory, focusing on both reducing hindrance job demands and increasing job resources. Each recommendation targets critical aspects of autonomy, competence, and relatedness, providing practical guidance for creating a more supportive learning environment.

### Tip 1. Reduce rigid curricular structures

Medical curricula often impose rigid, one-size-fits-all frameworks that limit learners' autonomy, which is essential for intrinsic motivation and well-being ^11^. By providing learners with more input into their schedules, clinical rotations, and electives, institutions can enhance their sense of ownership over their educational journey, allowing them to better align their experiences with personal interests and career goals. For instance, it is important to avoid controlling structures—such as mandatory wellness activities that do not have a clear purpose, and formal assessments of learners’ wellness—since these elements can undermine autonomy ^12^. Additionally, more flexible schedules, and allowing learners to have a say in rotation locations or specialty types can alleviate feelings of helplessness caused by rigid expectations, thereby increasing engagement ^13^. Implementing these changes may face resistance from faculty and administrators who are accustomed to traditional systems. To overcome this, pilot programs in smaller cohorts can demonstrate the positive effects of learner autonomy, and discussions with stakeholders can help highlight the benefits of flexible structures in fostering long-term well-being and reducing burnout.

### Tip 2. Limit high-stakes assessments

The reliance on high-stakes assessments, such as summative exams, often contributes to competence frustration, perfectionism, and anxiety among learners ^14^. These assessments typically prioritize summative evaluations over formative feedback, limiting opportunities for learners to gauge their progress or develop mastery. To address this, programs should shift focus from high-stakes exams to ongoing, formative assessments that provide learners with actionable insights into their strengths and areas for improvement. This approach fosters a sense of competence and growth, alleviating anxiety while promoting skill development. Implementing more holistic assessments, such as competency-based evaluations or portfolio-based learning, can also reduce stress and provide learners with a clearer picture of their development ^15^. Faculty may resist this shift due to their reliance on traditional exam-based evaluation, but incremental changes, like introducing more formative assessments and highlighting their impact on learner engagement, can help build buy-in for this approach.

### Tip 3. Minimize hierarchical and competitive dynamics

Hierarchical structures and competitive dynamics in medical training often lead to fear and feelings of alienation, as learners feel their worth is tied to external validation and performance metrics ^16^. This creates a culture where learners are less likely to express their authentic selves or engage in collaborative learning ^17^. Reducing hierarchical power differentials and fostering a more inclusive, collaborative environment is critical for promoting well-being. By shifting from a focus on individual achievement to collective growth, institutions can encourage learners to feel valued for their unique contributions, fostering stronger social connections and reducing isolation. In practical terms, this means promoting teamwork over competition and ensuring that learners are supported by faculty in ways that respect their individuality and unique strengths. To overcome resistance to this change, it is crucial to invest in faculty development that emphasizes the importance of collaboration and the negative impact of competitive pressures on learner well-being.

### Tip 4. Eliminate controlling faculty behaviours

Controlling faculty behaviours, such as micromanaging or maintaining rigid expectations, undermine learners’ autonomy and self-determination. When learners are pressured to conform to strict standards rather than being trusted to explore their own interests, and to adapt their learning styles, their intrinsic motivation and well-being suffers ^18^. Faculty should be trained to adopt a more autonomy-supportive teaching style, providing learners with the flexibility to make decisions and take ownership of their learning ^6^. Shifting from a directive, controlling approach to a facilitative one helps learners thrive by fostering self-regulated learning, allowing learners to build confidence in their abilities. Faculty may be resistant to this change, particularly if they are accustomed to a more structured, top-down teaching style. However, faculty development programs focused on autonomy-supportive teaching, combined with feedback from learners about the effectiveness of these methods, can highlight the reciprocal benefits to teacher of making this shift ^19^.

### Tip 5. Simplify administrative and bureaucratic processes

The complex administrative and bureaucratic processes in medical education can be a significant source of stress and frustration for learners, detracting from time and energy that could otherwise be spent on learning and clinical development ^20^. Medical programs should aim to streamline administrative tasks, such as reducing redundant documentation and eliminating unnecessary reporting, to alleviate cognitive load and support learners in focusing on their educational goals. Simplifying “tick box” processes related to Entrustable Professional Activities, for example, can save time and mental energy ^21^. Additionally, ensuring that learners have easy access to administrative support when needed can alleviate stress and help them feel more competent in managing their responsibilities ^20^. Resistance to change may stem from institutional inertia, but identifying redundancies and implementing tech-driven solutions, like digital platforms for documentation, can provide clear, tangible benefits in terms of efficiency and reduced stress, making it easier to garner support for these changes.

### Tip 6. Reduce unmanageable workload pressures

Unmanageable workload demands, including long hours, high patient loads, and excessive administrative tasks, are major contributors to burnout and emotional exhaustion ^22^. These pressures leave little room for self-care or personal growth, undermining learners' autonomy, competence, and relatedness. To mitigate this, medical programs should reduce excessive workload pressures by limiting on-call hours, reducing administrative duties, and ensuring that clinical responsibilities are manageable. Regular assessment of workload expectations is key to maintaining a sustainable balance between training and well-being. While concerns about patient care continuity or resource limitations may arise, engaging faculty and administrators in discussions about creating workload models that balance educational needs with learner well-being is essential. Pilot programs that reduce non-essential work demands can serve as proof of concept, helping to build consensus and support for long-term changes that prioritize learners' well-being.

## Structural supports: enhancing resources that satisfy basic psychological needs

Medical education must focus not only on reducing job demands that frustrate autonomy, competence, and relatedness, but also on introducing job resources and supports that proactively nurture these basic psychological needs. While the journey to becoming a physician is rich with opportunities for deep meaning, connection, and personal growth, these positive experiences do not happen automatically. It is essential to create the right conditions to foster these outcomes. The following section explores key ways of doing that, which can help learners maintain resilience, enhance well-being, and buffer against burnout.

### Tip 7. Balance learning and responsibility: involve learners in meaningful clinical work

To mitigate burnout, some programs have implemented policies that restrict learners' involvement in clinical service delivery, focusing their roles solely on education. While this aims to reduce stress, it can unintentionally undermine their sense of self-determination. When learners are limited to menial tasks, such as scut work (e.g., entering orders, filling out forms, coordinating hospital discharges) or observing rather than participating, they may feel a loss of control over their work (diminished autonomy) and disconnected from the real challenges of patient care (reduced competence). This can also erode their sense of purpose and contribution to the team (weakened relatedness). Instead of removing clinical responsibility, programs should ensure that learners are supported with adequate resources to help them balance educational and clinical demands. Involving learners in decision-making and giving them more opportunities to directly engage with patient care helps foster a sense of ownership, skill development, and connection to the broader healthcare mission ^23^. This not only prepares them for independent practice but also supports their well-being by addressing their core psychological needs.

### Tip 8. Provide learner-driven pathways to support autonomy

Autonomy-supportive learning environments are fundamental to fostering intrinsic motivation and engagement in medical education ^24^. Medical programs should design curricula that provide learners with greater choice and flexibility, allowing them to select electives, clinical rotations, and research opportunities that align with their interests and career goals. Doing these things not only enhances learners' sense of agency and motivation but also reduces stress by giving them more control over their educational trajectory. Although some faculty may resist the idea of increased autonomy due to concerns about standardization, pilot programs in smaller cohorts can help demonstrate the positive impact of autonomy on learner engagement and well-being. Importantly, autonomy should not be confused with independence; it is about providing meaningful rationales and structured opportunities for learners to make meaningful choices within the curriculum, supported by faculty guidance ^25^.

### Tip 9. Establish competence-building initiatives with personalized feedback

To foster learners' sense of competence, medical programs should integrate personalized feedback systems that go beyond traditional summative assessments. Competency-based education, combined with tailored, formative feedback, ensures that learners receive specific, actionable guidance on their progress, which enhances their sense of mastery and builds self-confidence ^26^. Peer assessments and simulation-based learning offer learners low-stakes environments to refine their skills and receive constructive feedback. Celebrating small achievements along the way also boosts self-efficacy, reducing feelings of inadequacy. Faculty may face time constraints in providing personalized feedback, but technology solutions, such as digital portfolios, can facilitate regular check-ins and streamline the feedback process, ensuring that learners receive continuous developmental support without overwhelming faculty workloads.

### Tip 10. Foster relatedness by building inclusive, supportive learning environments

Fostering relatedness is crucial for medical learners' well-being, and medical schools should prioritize creating supportive environments that embrace diversity and ensure all learners feel seen, valued, and respected ^27^. This includes dismantling systemic barriers to inclusion and promoting a culture that reflects the richness of diverse perspectives. Schools should intentionally design spaces where learners can contribute their unique voices and collaborate without fear of exclusion ^28^. Additionally, integrating inclusive practices into the curriculum (e.g., addressing implicit biases and creating opportunities for marginalized groups to lead) can help foster a sense of belonging and reduce feelings of alienation. Faculty development programs on equity, diversity, and inclusion (EDI) are key to addressing these systemic issues and ensuring that diversity is woven into the fabric of the learning environment, rather than treated as an afterthought.

### Tip 11. Encourage faculty-learner partnerships to promote collaborative learning

Long-term, collaborative faculty-learner partnerships are key to fostering a supportive learning environment. Longitudinal integrated clerkships (LICs), where learners remain with the same faculty or clinical team over extended periods, offer an effective model for fostering deeper connections and providing consistent feedback ^29^. Even if full LICs are not feasible, schools can adopt elements of this model by ensuring more consistent faculty pairings or relationship-centered rotations. This continuity allows learners to feel more supported, build trust with mentors, and receive ongoing feedback that strengthens their competence. Faculty workload concerns can be addressed by emphasizing the long-term benefits of stable, continuous relationships, both for learners' development and faculty satisfaction. Regular check-ins that focus not only on academic progress but also on emotional well-being can help deepen the connection between faculty and learners, fostering a more personalized and supportive learning environment.

### Tip 12. Embed wellness education and self-regulation into the curriculum

Rather than treating wellness as a standalone initiative, wellness education should be integrated into the medical curriculum to equip learners with tools for managing their psychological health. Embedding practices like mindfulness, emotion regulation, and self-reflection into core training helps learners identify when their basic psychological needs are being frustrated and gives them tools to cope with those challenges ^12^. Additionally, offering opt-out programs for counseling, peer-support groups, and wellness centers normalizes mental health support, which has been shown to increase engagement and help ensure that these resources are readily available when needed ^30^. This proactive approach encourages learners to prioritize their own well-being and view it as an essential element for long-term career sustainability. Embedding wellness strategies into the curriculum reduces the risk of burnout by teaching learners to manage stress and cultivate resilience as an ongoing part of their development.

## Conclusions

Addressing physician well-being through systemic reform requires a shift from isolated, individual-focused interventions to comprehensive, theory-guided approaches that target the structural drivers of burnout. By applying principles from Self-Determination Theory (SDT) and the Job Demands-Resources (JD-R) theory, this paper has outlined 12 practical tips to guide medical educators and institutions in creating environments that foster the satisfaction of learners' core psychological needs—autonomy, competence, and relatedness—while reducing the systemic barriers that frustrate these needs. The recommendations emphasize the importance of reforming rigid curricula, reducing stress-inducing assessments, fostering authentic faculty-learner partnerships, and enhancing career exploration opportunities, all aimed at increasing learner engagement, motivation, and well-being. By focusing on systemic changes that align with intrinsic motivation, educational institutions can cultivate physicians who not only thrive in their training but also sustain their well-being throughout their careers. These evidence-based, theory-driven reforms offer a clear pathway to building a healthier, more sustainable medical workforce.

## Ethics and consent

Ethical approval and consent were not required.

## Data Availability

No data are associated with this article.
