# Flourishing by Design: Applying Self-Determination Theory and the Job Demands-Resources Model to Systems-Level Wellness in Medical Education

**DOI:** 10.12688/mep.20886.2

**Published:** 2025-11-03

**Authors:** Adam Neufeld

**Affiliations:** 1 Department of Family Medicine, University of Calgary, Calgary, Alberta, Canada

**Keywords:** physician wellness, burnout prevention, Self-Determination Theory (SDT), Job Demands-Resources (JD-R) Theory, medical education reform, basic psychological needs

## Abstract

**Background:**

Physician burnout remains a defining challenge in medical education, driven by excessive demands and fragmented wellness initiatives. While calls for systemic reform grow louder, many efforts lack a unifying framework capable of addressing both distress and the cultivation of professional fulfillment.

**Methods:**

This guide applies a dual-theory lens—Self-Determination Theory (SDT) and the Job Demands–Resources (JD-R) model—to propose a systems-based approach to motivation and wellness. Drawing on empirical evidence and applied experience, it presents twelve actionable strategies across three ecological domains: the built environment, policy frameworks, and interpersonal dynamics. The first six strategies target hindrance demands that frustrate psychological needs and contribute to burnout; the next six strengthen resources that satisfy those needs and foster engagement, resilience, and well-being.

**Results:**

The strategies offer flexible, theoretically grounded entry points for reform. Rather than prescribing rigid solutions, they aim to support institutions in cultivating sustainable, human-centered learning environments where wellness is embedded—not bolted on. Examples include prioritizing formative over high-stakes assessments, making justice and safety integral to institutional design, and balancing clinical responsibility with developmental support.

**Conclusions:**

Integrating SDT and JD-R provides a rigorous, coherent, and scalable foundation for systems-level wellness initiatives. It reframes well-being not as the absence of burnout but as the presence of thriving—offering a shared language, validated metrics, and a roadmap for lasting cultural and structural transformation in medical education.

## Introduction

Burnout among medical trainees and physicians is no longer a background concern—it has become a defining feature of modern medical training. Despite years of wellness initiatives, distress persists, driven by excessive demands and insufficient systemic support ^1^. Institutional responses, though well-intentioned, often remain fragmented and reactive, lacking a coherent foundation for sustained, meaningful change.

Momentum is building for something more transformative. Reports such as *Revealing the Blind Spots and others* have exposed the structural barriers that continue to undermine physician wellness, calling for a shift from surface-level fixes to deeper, systemic reform ^2– 6^. Increasingly, scholars and educators argue that progress requires frameworks capable of addressing the full human experience—the light and the dark ^7^. Reducing burnout and distress is only half the task; fostering vitality, purpose, and joy is the other.

Self-Determination Theory (SDT) and the Job Demands-Resources (JD-R) model together offer such a framework. Each provides a distinct yet complementary lens: SDT explains how social and organizational contexts shape human motivation and wellness, while JD-R situates these processes within the structural realities of work. Their integration illuminates how institutional design can either nourish or deplete engagement, resilience, and professional growth—clarifying not only why people burn out, but how they can thrive.

This paper applies these frameworks to propose a systems-based approach to motivation and wellness in medical education. It outlines twelve interrelated strategies for reducing hindrance demands that frustrate psychological needs and for enhancing resources that satisfy them. These recommendations are grounded in theory and evidence and further informed by my work as a physician, educator, and SDT researcher collaborating with organizations to translate motivation science into practice. Rather than offering fixed prescriptions, they are presented as adaptable entry points for ongoing, context-sensitive reform—aimed at cultivating human-centered, sustainable environments for learning and care.

## Self-Determination Theory (SDT)

Self-Determination Theory (SDT) is a well-established, empirically validated framework for human motivation and well-being, applied extensively across education, healthcare, and organizational contexts ^8^. It identifies three basic psychological needs—autonomy (a sense of volition and agency), competence (a sense of capability and growth), and relatedness (a sense of belonging and respect)—as essential nutrients for thriving ^9^. When these needs are supported, individuals experience vitality and engagement; when they are frustrated, motivation deteriorates, leading to disengagement, burnout, and attrition.

Importantly, low need satisfaction is not the same as need frustration. SDT’s dual-process model distinguishes these pathways: need satisfaction predicts flourishing and resilience, while need frustration predicts strain, defensive coping, and ill-being ^10^. Thus, well-being and burnout are not opposites—they can co-exist. A physician may find deep meaning in their work yet feel depleted; another may feel competent but unseen. Recognizing this duality invites a more nuanced and dialectical view of wellness—one that understands flourishing not as the absence of hardship, but as the presence of support that allows people to grow through it.

SDT also clarifies a pervasive misconception in medicine—that autonomy equates to independence. Autonomy instead refers to acting with volition and alignment with personal values. Learners can feel autonomous even in highly structured or supervised settings when they are respected, informed, and meaningfully involved. Conversely, excessive “freedom” within unsupportive or isolating climates can feel alienating. Misunderstanding this distinction has long shaped medicine’s hidden curriculum, where stoicism, self-sacrifice, and solitary endurance are valorized over collaboration, mutuality, and supported growth ^11^. Correcting this cultural inheritance requires more than resilience training—it requires structural attention to how learning environments meet or thwart psychological needs.

## The Job Demands-Resources (JD-R) model

The Job Demands-Resources (JD-R) model offers a complementary, system-level framework for understanding workplace stress, engagement, and performance ^12^. At its core, the model posits that the balance between demands and resources determines whether individuals spiral toward engagement or exhaustion. When resources outweigh pressures, people experience energy, meaning, and growth; when demands chronically exceed support, motivation falters and burnout becomes predictable. JD-R reframes burnout not as individual weakness but as a systemic imbalance between effort and recovery.

Within this framework, workplace characteristics are categorized as demands (e.g., workload, emotional strain, bureaucracy) or resources (e.g., autonomy, feedback, mentorship) ^12^. Crucially, not all demands are harmful. *Challenge demands*, such as complex clinical cases or steep but achievable learning curves, can promote mastery and growth when matched with adequate support. *Hindrance demands*—avoidable stressors like redundant documentation, unclear expectations, or rigid hierarchies—drain energy, impede learning, and frustrate basic psychological needs.

In medical education, these dynamics are visible in rotation design, assessment systems, and supervision structures. Entrustment processes can become demotivating when perceived as inconsistent or bureaucratic, while performance pressure, emotional labour, and fragmented feedback loops often function as hindrance demands when left unbuffered. A review by Tummers *et al.* ^13^ found that job redesigns emphasizing autonomy, feedback, and team-based support significantly improved engagement and reduced strain. Applied to medical training, JD-R invites educators to ask not only what challenges learners face, but whether those challenges are energizing or depleting, and whether the system equips them to succeed.

## SDT and JD-R: A dual theory systems lens

SDT and JD-R together offer a unified framework linking individual motivation to system design. SDT explains how social contexts influence engagement through the satisfaction or frustration of basic psychological needs, while JD-R situates these processes within the structural ecology of work ^8, 14^. Integrating the two creates a dual-process lens that connects personal experience with organizational function, revealing how both well-being and burnout are cultivated, or constrained, by design.

Large-scale evidence supports this synthesis. In a study of nearly 2,000 educational leaders, Marsh *et al.* ^15^ found that job demands predicted need frustration, while resources predicted need satisfaction, with autonomy frustration emerging as the strongest predictor of burnout and intent to leave. Similar findings in higher education demonstrate that JD-R-informed interventions can reduce burnout and enhance engagement by recalibrating the balance between demands and supports ^16^. These results affirm that SDT and JD-R are not abstract theories but practical guides, clarifying both the roots of distress and the levers for sustainable motivation and flourishing.

The twelve strategies that follow draw from these frameworks and from lived experience. The first six target hindrance demands that frustrate psychological needs; the next six amplify resources that satisfy them. Each serves as a flexible entry point for reform, adaptable across settings and scales. Collectively, they form a blueprint for aligning policies, practices, and culture with what truly sustains learner and physician well-being.

While each strategy highlights a specific organizational lever, these levers are nested within broader systemic forces. The built environment—clinical work areas, call rooms, lounges, and teaching spaces—shapes motivation and wellness in subtle but powerful ways. Layout, lighting, noise, privacy, and access to rest or daylight all affect psychological safety, fatigue, and perceived value ^17^. Policy frameworks, like scheduling structures, reporting processes, and workload limits, determine how demands are distributed and how autonomy is enabled or constrained. Interpersonal dynamics, including trust, respect, and feedback quality, mediate the day-to-day experience of learning. These domains interact continuously: even the most supportive teacher may struggle to meet learner needs in a chaotic, understaffed, or poorly designed system. Recognizing these intersections allows institutions to move beyond piecemeal interventions toward integrated, design-level change, where the system itself becomes an ally in motivation and well-being.

## Reducing hindrance demands: Addressing the frustration of basic psychological needs

To reduce burnout, medical institutions must first confront the systemic conditions that frustrate basic psychological needs. These conditions act as hindrance demands—structural barriers that drain energy, limit autonomy, undermine competence, and thwart relatedness. The following strategies target six common, remediable sources of need frustration.

### Tip 1. Loosen curricular rigidity to restore learner autonomy

Rigid, one-size-fits-all curricula strip learners of agency, leaving little room for input into schedules, rotations, or electives ^18^. When preferences are solicited by rarely honoured, or when final schedules arrive only days before rotations, autonomy is quietly thwarted. Compliance-focused communication amplifies the message that structure matters more than growth, reducing learners to passive recipients rather than active participants in their own development.

Evidence confirms the risk of rigidity. Hansen *et al.* ^19^ found that motivation shifted from controlled to autonomous when scheduling and transitions aligned with SDT principles, whereas externally imposed structures caused motivational setbacks. Likewise, Liu *et al.* ^20^ and Zhu *et al.* ^21^ showed that autonomy-supportive curricular redesigns enhanced motivation and reduced stress.

Programs can restore ownership through flexible scheduling, elective choice, and individualized pathways, transforming training from a conveyor belt into a journey where learners feel included, capable, and engaged. When flexibility is not feasible, autonomy can still be supported through transparent rationales and opportunities for input. Even fixed expectations can be internalized when learners understand their purpose and relevance ^22^. Meaningful involvement, not necessarily choice, is what fosters motivation and belonging.

### Tip 2. Prioritize formative over high-stakes assessment

Cultures dominated by scores, rankings, and constant evaluation push learners to perform for approval rather than mastery, fueling impression management, impostorism, and burnout ^23, 24^. In such climates, help-seeking becomes risky: disclosure feels career-limiting, and mistakes are hidden instead of explored ^25^. From a JD-R perspective, perpetual evaluative pressures act as a hindrance demand that drains energy and frustrates competence. The result is defensive learning—an orientation toward avoiding failure rather than pursuing growth.

Formative, developmental approaches can reverse this trajectory. Programmatic assessment—portfolios, frequent low-stakes observations, and structured reflection—encourages learners to engage openly with feedback and track their progress over time ^26, 27^. These methods position learning through assessment rather than after it, fostering both autonomy and competence. Evidence consistently shows that mastery-oriented goals strengthen resilience and self-regulation, whereas performance goals heighten anxiety and disengagement ^28^. When institutions shift their emphasis from summative outcomes to formative dialogue, assessment becomes a catalyst for curiosity, reflection, and self-directed growth. The ultimate goal of assessment is not to prove competence, but to build it.

### Tip 3. Flatten hierarchies and diminish competitive climates

Hierarchical and competitive environments fundamentally shape how learners relate to their work and to one another. When contexts support choice, self-regulation, and genuine interest-taking, learners adopt an autonomous orientation that fosters engagement and growth. When they instead pressure compliance or feel unpredictable and beyond influence, anxiety, helplessness, and disengagement follow. Neufeld *et al.* ^29^ found that residents perceiving autonomy reported markedly less impostorism and burnout, while Salehi *et al.* ^30^ showed that entrenched hierarchies and competition fuel mistreatment, moral distress, and silence—especially in procedural contexts.

These hierarchies also perpetuate a culture of suppression—cultural artifacts in which stoicism and silence are mistaken for professionalism. Suppression and eventual dysregulation are not signs of fragility but natural responses to environments that feel unsafe for emotion. Over time, these adaptations impede trust and relatedness, sustaining disengagement and ill-being. True psychological safety depends not on erasing emotion but on climates that allow it to be acknowledged, shared, and integrated. When learners can process and find meaning in difficult experiences—grief, fear, shame, and moral distress—they build empathy, resilience, and authenticity ^31^.

Programs can counteract these effects by re-distributing authority, emphasizing team-based goals, and rewarding collaboration over comparison ^32– 34^. When learners experience genuine voice and psychological safety, they are more willing to share uncertainty and engage in collective problem-solving. Otherwise, intrinsic motivation—driven by curiosity and shared purpose—cannot take root in climates where status eclipses belonging. From an SDT/JD-R perspective, dismantling rigid hierarchies converts harmful hindrance demands into relational resources that foster trust, and inclusion. Faculty development that cultivates emotional integration, coupled with assessment of emotion regulation and expression as indicators of safety rather than individual deficit, can illuminate where fear or shame persist and guide systemic repair ^31^.

### Tip 4. Replace controlling faculty behaviours with autonomy support

Micromanagement, rigid directives, and public shaming do more than demoralize learners—they actively frustrate autonomy, competence, and relatedness, triggering stress, self-protection, and withdrawal ^35^. Large-scale, multi-site studies show that when faculty fail to support these psychological needs, residents report higher burnout, disengagement, and weaker organizational commitment ^36^. Within SDT’s dual-process model, this dynamic represents the “dark pathway” of motivation, where pressure and fear, rather than volition and purpose, drive behaviour. 

Breaking this cycle requires more than isolated workshops. Controlling behaviours are rarely malicious; they often stem from faculty members’ own need frustration or from cultural norms that equate control with rigour and efficiency ^37, 38^. Addressing them therefore demands institutional (not just individual) change. Autonomy-supportive strategies—acknowledging perspectives, offering rationales, and inviting input—should be woven into faculty development, feedback, and evaluation systems. Just as importantly, institutions must care for faculty well-being itself: unsupported educators are more likely to teach in controlling ways. When leaders model empathy, flexibility, and reflective feedback, they demonstrate that control is not synonymous with competence. Even small relational shifts—reframing feedback as collaboration rather than correction—can rebuild trust and rehumanize the learning environment ^39^. Over time, these practices replace coercive compliance with shared purpose, transforming both teaching and learning into intrinsically motivating, mutually reinforcing processes.

### Tip 5. Streamline bureaucracy to reduce cognitive and emotional load

Excessive workload and administrative burden remain among the strongest predictors of burnout in medicine ^40^. For learners, this extends beyond clinical care to include documentation, assessments, and mandatory modules that often feel duplicative or disconnected from purpose. Such hindrance demands drain energy and undermine autonomy and competence by diverting effort from meaningful learning. Large-scale workforce studies confirm that when systems are weighed down by bureaucracy and insufficient support, engagement and satisfaction decline while burnout accelerates ^41^.

In medical education, entrustment and assessment systems often feel performative, with feedback reduced to paperwork and checkboxes ^42, 43^. Fragmented IT systems and redundant forms overload cognition and signal mistrust, while poorly designed physical spaces—overcrowded nurse stations, inadequate work surfaces, harsh lighting, and noise—compound fatigue and interfere with focus. Learners documenting while standing in hallways or competing for computers internalize a quiet message—that their time and attention are expendable.

Programs can consolidate digital platforms, eliminate duplication, and simplify EPA workflows ^44^. Reliable, private workstations and quiet documentation zones, equipped with adequate seating, technology, and lighting, enhance both cognitive efficiency and psychological safety. Even modest design changes, like reducing hallway documentation or optimizing workspace layout, convey institutional respect and restore a sense of competence. Providing protected time and on-demand administrative support shifts culture from compliance to contribution. When bureaucratic processes align with learning goals, they cease to be a drain and become a shared resource for clarity, coordination, and meaning.

### Tip 6. Make justice and safety integral to institutional design

Sexual harassment, abuse, and discrimination remain alarmingly prevalent in medicine, with systematic reviews reporting rates as high as 60%, particularly among women and marginalized groups ^45^. Yet institutional responses often compound harm—processes are opaque, overly procedural, and more attuned to risk management than to healing. As Siad and Rabi observe, reports are frequently miscategorized or reframed, with greater attention paid to institutional liability than to the dignity and safety of those affected ^46^. A learner who reports a supervisor’s inappropriate behaviour may see the case downgraded to “incivility,” delaying protection and deepening disillusionment. What appears administrative is often experienced as silencing.

From an SDT standpoint, these systems do more than fail ethically—they frustrate core psychological needs. JD-R positions such obstructive climates as hindrance demands that deplete energy, foster mistrust, and create moral injury. When people fear reprisal or futility, they disengage, not because they are fragile, but because the system itself feels unsafe to participate in.

Programs can begin to reverse this by embedding justice directly into design. Transparent reporting pathways, trauma-informed support, and meaningful choice in how individuals engage restore autonomy and trust ^47^. This includes closing feedback loops, involving trained advocates, and tracking patterns across departments rather than isolating cases. Regular policy review with learner and faculty input ensures that procedures reflect lived experience, not just legal compliance. When institutions deliver justice with humanity—centering respect, clarity, and care—they transform accountability into a motivational resource that rebuilds belonging, integrity, and hope.

## Amplifying resources: supporting the satisfaction of basic psychological needs

Medicine is inherently rich with opportunities for meaning, mastery, and connection, but systemic inefficiencies and cultural norms often obstruct these resources. Enhancing wellness therefore means not only removing barriers but cultivating the supports that nourish motivation. The following strategies strengthen autonomy, competence, and relatedness—the essential psychological nutrients for thriving in demanding environments.

### Tip 7. Design workloads that replenish energy and reinforce purpose

When designed intentionally, workload becomes a vehicle for learning and identity formation rather than a source of depletion. Within JD-R, challenge demands such as steep learning curves, clinical responsibility, and complex patient care can be deeply motivating when balanced by resources like attentive supervision, timely feedback, and recovery time. What distinguishes growth from strain is not the number of hours worked, but whether those hours occur within predictable, supported, and educationally balanced systems ^48^. Evidence from Jung *et al.* ^49^ shows that engagement can buffer the impact of heavy workloads, turning demanding experiences into opportunities for development rather than depletion.

Resources that sustain energy include predictable schedules, protected rest, manageable cognitive load, and opportunities for meaningful contribution. Even small design choices—quiet call rooms, adequate lighting, restorative spaces—shape focus and recovery. Completing a patient task from start to finish or receiving authentic appreciation reinforces competence and relatedness, re-anchoring effort to purpose. Conversely, excessive cross-coverage, redundant paperwork, and unacknowledged labour turn challenge into hindrance, draining motivation.

Programs can promote sustainable excellence by embedding recovery buffers, distributing service equitably, and making purpose visible through reflection, storytelling, and role modelling. When work is designed not as endurance but as evolution, learners experience effort as energizing rather than depleting—a defining feature of need satisfaction and self-determined motivation.

### Tip 8. Balance clinical responsibility with developmental support

Authentic patient care—not peripheral tasks or busy work—nourishes autonomy, competence, and relatedness. Yet in well-intended efforts to protect wellness, some programs inadvertently strip responsibility away, treating all difficulty as harmful. From a JD-R perspective, this is a category error: many responsibilities are challenge demands that stretch learners and strengthen motivation when paired with adequate support ^50^. Removing them can backfire, depriving learners of the very sense of mastery and purpose that sustains well-being. Conversely, excessive oversight creates its own harms—Lee *et al.* ^51^ found that micromanagement consistently diminished autonomy, strained relationships, and even compromised patient care.

The goal is calibration, not protection. Sawatsky *et al.* ^52^ showed that entrustment aligned with competence strengthened engagement and professional identity, allowing learners to internalize responsibility as growth rather than burden. A resident trusted to lead a patient handover under guided supervision experiences challenge as empowerment, not exposure. Programs can apply a simple litmus test before policy or workflow change: *Does this remove low-value hindrance, preserve meaningful challenge, and ensure adequate support for both faculty and learners?* When responsibility is scaffolded—not withheld or micromanaged—it becomes a renewable source of confidence, connection, and intrinsic motivation.

### Tip 9. Elevate learner voice through authentic partnership

Many institutions celebrate learner leadership, yet participation often stops at tokenism—decisions are made, and learners are merely informed. Such symbolic involvement offers voice without influence, frustrating autonomy and undermining trust. Reviews confirm this gap: engagement in decision-making is inconsistently defined, limited in scope, and prone to performative consultation ^53^. Learners frequently describe the dissonance of being “involved” but not empowered, with entrenched hierarchies constraining their reach ^54^. When this happens, potentially energizing initiatives—curricular reform, technology adoption, or social accountability projects—become hindrance demands that deplete energy instead of fueling resolve.

Authentic partnership looks different. Evidence from student-faculty co-design demonstrates that genuine collaboration—where learners share accountability and influence— enhances relatedness, competence, and autonomy, while strengthening collective resilience ^55^. Programs can deepen authenticity by (1) creating structures with institutional memory, (2) ensuring input precedes decisions rather than follows them, and (3) aligning opportunities with both learner growth and institutional priorities. Learners today are driving advances in equity, digital innovation, and climate-conscious medicine, but adoption remains fragmented ^56^. Concrete examples, from student-led curriculum redesigns to assessment reforms, show how authentic partnership restores trust, cultivates belonging, and turns participation into genuine educational progress ^57^.

### Tip 10. Deliver feedback that builds competence and agency

High frequency, low-stakes feedback—through bedside observation, coaching conversations, or reflective portfolios—helps learners calibrate performance and build mastery. Programs that integrate brief goal-setting and reflection report higher engagement and more accurate self-assessment than those relying on sporadic input ^58^. Many educators struggle with “negative” feedback, equating critique with harm; yet within SDT, even corrective feedback can enhance motivation when offered with empathy, clarity, and rationale ^59^. Framing feedback as guidance rather than judgment transforms anxiety into trust and reinforces competence and internalization. Within the JD-R model, these practices recast feedback from a performance threat into a protective resource that promotes confidence, learning, and resilience.

When feedback is inconsistent, bureaucratic, or framed as evaluation rather than growth, it becomes a hindrance demand, fueling anxiety and disengagement. A vital but often overlooked antidote is agentic engagement: the learner’s proactive role in shaping feedback ^60^. Defined as contributing ideas, clarifying goals, and inviting critique, agentic engagement predicts stronger motivation, persistence, and learning outcomes ^61, 62^. When learners articulate goals and faculty respond with curiosity—“Let’s plan your next attempt together”—feedback becomes a dialogue rather than a verdict. Preparing both parties for this kind of exchange ensures that assessment is collaborative, cultivating not only competence but a shared sense of agency and progress ^63, 64^.

### Tip 11. Foster belonging through inclusion and psychological safety

Training environments must make learners feel safe, seen, and valued. This includes both social and physical spaces: inclusive imagery, accessible design, and welcoming communal areas can signal belonging as powerfully as any policy. When learners can bring their full selves to training, relatedness deepens, competence grows, and motivation strengthens ^65^. Within the JD-R framework, inclusion and psychological safety function as core job resources that buffer stress and sustain engagement ^66^.

Evidence consistently supports this. Ganotice *et al.* ^67^ found that curricula explicitly supporting autonomy, competence, and relatedness improved teamwork, engagement, and collective goal attainment. McClintock *et al.* ^68^ showed that autonomy supportive behaviours—clear expectations, trust, and genuine interest—were critical to psychological safety, while disinterest or dismissiveness rapidly fractured it. Once safety was lost, learners rarely recalled it being restored, underscoring the need for proactive climate building. Simple acts, such as faculty acknowledging uncertainty or sharing their own learning missteps, can rebuild trust and signal humility in ways that policies alone cannot.

Programs can embed inclusion through anti-bias education, amplifying underrepresented voices, and creating leadership pathways for marginalized groups. Faculty development should emphasize empathy, humility, and reflective practice. Without these, diversity remains aspirational rather than embodied. Prioritizing relatedness is not only ethical—it is motivational infrastructure, turning learning environments into places where people can both belong and thrive ^66^.

### Tip 12. Build relational continuity through faculty-learner partnerships

Sustained, trusting faculty relationships are foundational to motivation, identity formation, and well-being. Longitudinal integrated clerkships (LICs), where learners remain with the same team or preceptor, provide continuity that strengthens feedback, coaching, and mentorship ^69^. A BEME systematic review found consistent benefits for learning, professional growth, and satisfaction ^70^. Even when full LICs are impractical, similar outcomes can be achieved through stable team assignments, consistent preceptors, or aligned schedules that preserve relational continuity.

These structures create space for deeper feedback, fairer assessment, and genuine emotional support ^58^. Over time, repeated exposure to consistent role models allows learners to internalize professional values and see themselves as developing physicians ^71^. Within the JD-R framework, such relationships operate as powerful job resources, reinforcing relatedness and enabling learners to stretch beyond comfort zones with security and trust. Faculty members who follow learners longitudinally can calibrate expectations more fairly, celebrate growth authentically, and model vulnerability, showing that expertise and humility can coexist.

Faculty workload concerns are legitimate, yet continuity benefits both sides ^72^. Mentors often report renewed meaning and connection when they can witness learners’ progression over time. Institutions can support these partnerships through protected mentorship time, faculty coaching, and deliberate pairing strategies. Investing in relational continuity transforms supervision from a series of isolated encounters into a developmental alliance—one that sustains motivation, deepens learning, and fosters mutual growth across the continuum of medical education.

## Conclusion: From theory to transformative practice

Medical education has made meaningful progress in addressing physician burnout, yet efforts often remain fragmented and overly focused on individual coping. In contrast, Self-Determination Theory (SDT) and the Job Demands-Resources (JD-R) model provide a coherent foundation for sustainable reform, clarifying not only why motivation matters, but how to cultivate it across systems. This paper outlined twelve interconnected strategies that reduce hindrance demands and strengthen resources, moving wellness efforts beyond surface-level fixes toward enduring cultural transformation. The goal is not to remove challenge but to ensure that high standards and hard work unfold within climates that support autonomy, competence, and relatedness.

Across the literature, countless interventions show promise but remain difficult to replicate and theoretically underpowered. SDT and JD-R unite these disparate efforts under a single explanatory framework, offering shared language, validated metrics, and a scalable roadmap for reform. Their integration enables educators and institutions to coordinate strategies, compare outcomes, and build cumulative knowledge grounded in motivation science rather than intuition. The scarcity of replication in physician wellness research likely reflects this lack of coherence and limited integration with well-established frameworks in organizational psychology—fields that have long examined how system design shapes human performance and well-being. By bridging these disciplines, medicine can move from experimentation to evidence-based reform.

Ultimately, the call to align systems with human needs is not only a matter of design—it is a matter of conscience. Physicians and learners cannot flourish in systems that drain their sense of purpose or isolate them from genuine connection. Supporting motivation is therefore a professional, ethical, and human imperative—a reminder that well-being and excellence arise from the same moral foundation. When institutions honour this truth—through the spaces they create, the policies they craft, and the relationships they sustain—medical education can move beyond endurance toward a culture of vitality, integrity, and compassion.

## Ethics and consent

Ethical approval and consent were not required.

## Data Availability

No data are associated with this article.
