# Flourishing by Design: Applying Self-Determination Theory and the Job Demands-Resources Model to Systems-Level Wellness in Medical Education

**DOI:** 10.12688/mep.20886.3

**Published:** 2026-01-20

**Authors:** Adam Neufeld

**Affiliations:** 1 Department of Family Medicine, University of Calgary, Calgary, Alberta, Canada

**Keywords:** physician wellness, burnout prevention, Self-Determination Theory (SDT), Job Demands-Resources (JD-R) Theory, medical education reform, basic psychological needs

## Abstract

**Background:**

Physician burnout remains a defining challenge in medical education, driven by excessive demands and fragmented wellness initiatives. While calls for systemic reform grow louder, many efforts lack a unifying framework capable of addressing both distress and the cultivation of professional fulfillment.

**Methods:**

This guide applies a dual-theory lens—Self-Determination Theory (SDT) and the Job Demands–Resources (JD-R) model—to propose a systems-based approach to motivation and wellness. Drawing on empirical evidence and applied experience, it presents twelve actionable strategies across three ecological domains: the built environment, policy frameworks, and interpersonal dynamics. The first six strategies target hindrance demands that frustrate psychological needs and contribute to burnout; the next six strengthen resources that satisfy those needs and foster engagement, resilience, and well-being.

**Results:**

The strategies offer flexible, theoretically grounded entry points for reform, supporting institutions in cultivating sustainable, human-centered learning environments where wellness is embedded—not bolted on. Examples include prioritizing formative over high-stakes assessments, integrating justice and safety into institutional design, and balancing clinical responsibility with developmental support.

**Conclusions:**

Integrating SDT and JD-R provides a rigorous, coherent, and scalable foundation for systems-level wellness initiatives. It reframes well-being not as the absence of burnout but as the presence of flourishing—offering a shared language, validated metrics, and a roadmap for lasting cultural and structural transformation in medical education.

## Introduction

Burnout among medical trainees and physicians is no longer a background concern—it has become a defining feature of modern medical training. Despite years of wellness initiatives, distress persists, driven by excessive demands and insufficient systemic support ^1^. Institutional responses, though well-intentioned, often remain fragmented and reactive, lacking a coherent foundation for sustained, meaningful change.

Momentum is building for something more transformative. Reports such as *Revealing the Blind Spots and* related national analyses have exposed the structural barriers that continue to undermine physician wellness, calling for a shift from surface-level fixes to deeper, systemic reform ^2– 6^. Increasingly, scholars and educators argue that progress requires frameworks capable of addressing both the negative and positive dimensions of human functioning—psychological distress and psychological growth ^7^. Reducing burnout and ill-being is only half the task. Fostering vitality, purpose, and joy is the other.

Self-Determination Theory (SDT) and the Job Demands-Resources (JD-R) model together offer such a framework. Each provides a distinct yet complementary lens: SDT explains how social and organizational contexts shape human motivation and wellness, while JD-R situates these processes within the structural realities of work. Their integration illuminates how institutional design can either nourish or deplete engagement, resilience, and professional growth—clarifying not only why people burn out, but how they can thrive.

This paper applies these frameworks to propose a systems-based approach to motivation and wellness in medical education. It outlines twelve interrelated strategies for reducing hindrance demands that frustrate psychological needs and for enhancing resources that satisfy them. These recommendations are grounded in theory and evidence and further informed by my work as a physician, educator, and SDT researcher collaborating with organizations to translate motivation science into practice. Rather than offering fixed prescriptions, they are presented as adaptable entry points for ongoing, context-sensitive reform—ones aimed at cultivating human-centered, sustainable environments for learning and care.

## Self-Determination Theory (SDT)

Self-Determination Theory (SDT) is a well-established, empirically validated framework for human motivation and well-being, applied extensively across education, healthcare, and organizational contexts ^8^. It identifies three basic psychological needs—autonomy (a sense of volition and agency), competence (a sense of capability and growth), and relatedness (a sense of belonging and respect)—as essential nutrients for thriving ^9^. When these needs are supported, individuals experience vitality and engagement; when they are frustrated, motivation deteriorates, leading to disengagement, burnout, and attrition.

Importantly, low need satisfaction is not the same as need frustration. SDT’s dual-process model distinguishes these pathways: need satisfaction predicts flourishing and resilience, while need frustration predicts strain, defensive coping, and ill-being ^10^. Thus, well-being and burnout are not opposites, but can co-exist. A physician may find deep meaning in their work but feel depleted; another may feel competent but unseen. Recognizing this duality invites a more nuanced and developmental view of wellness—one that understands flourishing not as the absence of hardship, but as the presence of support that allows people to grow through it.

SDT also clarifies a pervasive misconception in medicine—that autonomy equates to independence. Autonomy instead refers to acting with volition and alignment with personal values. Learners can feel autonomous even in highly structured or supervised settings when they are respected, informed, and meaningfully involved. Conversely, excessive “freedom” within unsupportive or isolating climates can feel alienating. Misunderstanding this distinction has long shaped medicine’s hidden curriculum, where stoicism, self-sacrifice, and solitary endurance are valorized over collaboration, mutuality, and supported growth ^11^. Correcting this cultural inheritance requires more than resilience training—it requires structural attention to how learning environments meet or thwart psychological needs.

## The Job Demands-Resources (JD-R) model

The Job Demands-Resources (JD-R) model offers a complementary, system-level framework for understanding workplace stress, engagement, and performance ^12^. At its core, the model posits that the balance between demands and resources determines whether individuals shift toward engagement or exhaustion. When resources outweigh pressures, people experience energy, meaning, and growth; when demands chronically exceed support, motivation falters and burnout becomes predictable. JD-R reframes burnout not as individual weakness but as a systemic imbalance between effort and recovery.

Within this framework, workplace characteristics are categorized as demands (e.g., workload, emotional strain, bureaucracy) or resources (e.g., autonomy, feedback, mentorship) ^12^. Crucially, not all demands are harmful. *Challenge demands*, such as complex clinical cases or steep but achievable learning curves, can promote mastery and growth when matched with adequate support. *Hindrance demands*—avoidable stressors like redundant documentation, unclear expectations, or rigid hierarchies—drain energy, impede learning, and frustrate basic psychological needs.

In medical education, these dynamics are visible in rotation design, assessment systems, and supervision structures. Entrustment processes can become demotivating when perceived as inconsistent or bureaucratic, while performance pressure, emotional labour, and fragmented feedback loops often function as hindrance demands when left unbuffered. A review by Tummers *et al*. ^13^ found that job redesigns emphasizing autonomy, feedback, and team-based support significantly improved engagement and reduced strain. Applied to medical training, JD-R invites educators to ask not only what challenges learners face, but whether those challenges are energizing or depleting, and whether the system equips them to succeed.

## SDT and JD-R: A dual theory systems lens

SDT and JD-R together offer a unified framework linking individual motivation to system design. SDT explains how social contexts influence engagement through the satisfaction or frustration of basic psychological needs, while JD-R situates these processes within the structural ecology of work ^8, 14^. Integrating the two creates a dual-process lens that connects personal experience with organizational function, revealing how both well-being and burnout are cultivated, or constrained, by design.

Large-scale evidence supports this synthesis. In a study of nearly 2,000 educational leaders, Marsh *et al.* ^15^ found that job demands predicted need frustration, while resources predicted need satisfaction, with autonomy frustration emerging as the strongest predictor of burnout and intent to leave. Similar findings in higher education demonstrate that JD-R-informed interventions can reduce burnout and enhance engagement by recalibrating the balance between demands and supports ^16^. These results affirm that SDT and JD-R are not abstract theories but practical guides, clarifying both the roots of distress and the levers for sustainable motivation and flourishing.

The twelve strategies that follow draw from these frameworks and from lived experience. The first six target hindrance demands that frustrate psychological needs; the next six amplify resources that satisfy them. Each serves as a flexible entry point for reform, adaptable across settings and scales. Collectively, they form a blueprint for aligning policies, practices, and culture with what truly sustains learner and physician well-being.

While each strategy highlights a specific organizational lever, these levers are nested within broader systemic forces. The built environment—clinical work areas, call rooms, lounges, and teaching spaces—shapes motivation and wellness in subtle but powerful ways. Layout, lighting, noise, privacy, and access to rest or daylight all affect psychological safety, fatigue, and perceived value ^17^. Policy frameworks, like scheduling structures, reporting processes, and workload limits, determine how demands are distributed and how autonomy is enabled or constrained. Interpersonal dynamics, including trust, respect, and feedback quality, mediate the daily experience of learning.

These domains interact continuously: even the most supportive teacher may struggle to meet learner needs in a chaotic, understaffed, or poorly designed system. Recognizing these intersections allows institutions to move beyond piecemeal interventions toward integrated, design-level change, where the system itself becomes an ally in motivation and well-being.

## Reducing hindrance demands: Addressing the frustration of basic psychological needs

To reduce burnout, medical institutions must first confront the systemic conditions that frustrate basic psychological needs. These conditions act as hindrance demands—structural barriers that drain energy, limit autonomy, undermine competence, and thwart relatedness. The following strategies target six common, remediable sources of need frustration.

### Tip 1. Loosen curricular rigidity to restore learner autonomy

Rigid, one-size-fits-all curricula strip learners of agency, leaving little room for input into schedules, rotations, or electives ^18^. When preferences are solicited by rarely honoured, or when final schedules arrive only days before rotations, autonomy is quietly thwarted. Compliance-focused communication amplifies the message that structure matters more than growth, reducing learners to passive recipients rather than active participants in their own development. These “small” constraints accumulate, signalling low voice and limited trust, and shifting motivation toward compliance rather than internalization.

Evidence confirms the risk of rigidity. Hansen *et al*. ^19^ found that learner motivation shifted from controlled to autonomous when scheduling and transitions aligned with SDT principles, whereas externally imposed structures caused motivational setbacks. Likewise, Liu *et al*. ^20^ and Zhu *et al*. ^21^ both showed that autonomy-supportive curricular redesigns enhanced motivation and reduced stress. When avoidable rigidity adds friction without adding learning value, it functions as a hindrance demand, consuming energy while offering little developmental return.

Programs can restore ownership through flexible scheduling, elective choice, and individualized pathways, transforming training from a conveyor belt into a journey where learners feel included, capable, and engaged. When flexibility is not feasible, autonomy can still be supported through transparent rationales and opportunities for input. Even fixed expectations can be internalized when learners understand their purpose and relevance ^22^. In practice, autonomy support often looks like predictable timelines, clear rationales, and real opportunities to shape schedules where feasible, so structure supports development rather than merely enforcing compliance.

### Tip 2. Prioritize formative over high-stakes assessment

Cultures dominated by scores, rankings, and constant evaluation push learners to perform for approval rather than mastery, fueling impression management, impostorism, and burnout ^23, 24^. In such climates, help-seeking becomes risky: disclosure feels career-limiting, and mistakes are hidden instead of explored ^25^. From a JD-R perspective, perpetual evaluative pressures act as a hindrance demand that drains energy and frustrates competence. The result is defensive learning—an orientation toward avoiding failure rather than pursuing growth. Over time, curiosity and self-directed development give way to surveillance, caution, and performance management.

Formative, developmental approaches can reverse this trajectory. Programmatic assessment—portfolios, frequent low-stakes observations, and structured reflection—encourages learners to engage openly with feedback and track their progress over time ^26, 27^. These methods position learning through assessment rather than after it, fostering both autonomy and competence. Evidence consistently shows that mastery-oriented goals strengthen resilience and self-regulation, whereas performance goals heighten anxiety and disengagement ^28^.

When institutions shift from ranking to coaching, assessment becomes a catalyst for curiosity and self-directed growth. Operationally, this means increasing high-frequency, low-stakes observation and reflection, while reserving high-stakes decisions for aggregated, longitudinal evidence.

### Tip 3. Flatten hierarchies and diminish competitive climates

Hierarchical and competitive environments fundamentally shape how learners relate to their work and to one another. When contexts support choice, self-regulation, and genuine interest-taking, learners adopt an autonomous orientation that fosters engagement and growth. When they instead pressure compliance or feel unpredictable and beyond influence, anxiety, helplessness, and disengagement follow. Neufeld *et al.* ^29^ found that residents perceiving autonomy reported markedly less impostorism and burnout, while Salehi *et al.* ^30^ showed that entrenched hierarchies and competition fuel mistreatment, moral distress, and silence—especially in procedural contexts. In JD-R terms, rigid hierarchy amplifies social threat and uncertainty while restricting access to relational resources, making day-to-day learning feel higher-stakes than it needs to be.

These hierarchies also perpetuate a culture of suppression, where stoicism and silence are mistaken for professionalism. In practice, this often looks like guarded help-seeking, avoidance of questions, and reluctance to name uncertainty—understandable adaptations in climates where mistakes carry social cost. True psychological safety depends on climates that allow emotion to be acknowledged, shared, and integrated. When learners can process and find meaning in difficult experiences—grief, fear, shame, and moral distress—they build empathy, resilience, and authenticity ^31^. A simple diagnostic for leaders is whether the local culture rewards honest uncertainty or penalizes it.

Programs can counteract these effects by re-distributing authority, emphasizing team-based goals, and rewarding collaboration over comparison ^32– 34^. When learners experience genuine voice and psychological safety, they are more willing to share uncertainty and engage in collective problem-solving. Otherwise, intrinsic motivation—driven by curiosity and shared purpose—cannot take root. Practically, leaders can lower unnecessary hierarchy by normalizing questions, modeling uncertainty, and designing team norms that make speaking up routine, so collaboration, not status management, becomes the default learning climate ^31^.

### Tip 4. Replace controlling faculty behaviours with autonomy support

Micromanagement, rigid directives, and public shaming do more than demoralize learners—they actively frustrate autonomy, competence, and relatedness, triggering stress, self-protection, and withdrawal ^35^. Large-scale, multi-site studies show that when faculty fail to support these needs, residents show higher burnout, disengagement, and weaker organizational commitment ^36^. Within SDT’s dual-process model, this dynamic represents the “dark pathway” of motivation, where pressure and fear, rather than volition and purpose, drive behaviour. Within JD-R, controlling supervision functions as a social-evaluative demand, making routine learning chronically effortful.

Breaking this cycle requires more than isolated workshops. Controlling behaviours are rarely malicious; they more often reflect faculty members’ own need frustration or entrenched norms that equate control with rigour and efficiency ^37, 38^. Addressing them therefore demands institutional change. Autonomy-supportive strategies—acknowledging perspectives, offering rationales, and inviting input—should be woven into faculty development, feedback, and evaluation systems. Institutions can also reduce the upstream pressures that elicit controlling habits (e.g., time scarcity, role overload, unclear expectations), making autonomy support more feasible in daily supervision.

When leaders model empathy, flexibility, and reflective feedback, they demonstrate that control is not synonymous with competence. Embedding autonomy support into routine practice—how feedback is delivered, how choices are framed, and how time pressures are managed—ensures that supervision reliably builds capacity and trust rather than fear and withdrawal ^39^.

### Tip 5. Streamline bureaucracy to reduce cognitive and emotional load

Excessive workload and administrative burden remain among the strongest predictors of burnout in medicine ^40^. For learners, this extends beyond clinical care to include documentation, assessments, and mandatory modules that often feel duplicative or disconnected from purpose. Such hindrance demands drain energy and undermine autonomy and competence by diverting effort from meaningful learning. Large-scale workforce studies confirm that when systems are weighed down by bureaucracy and insufficient support, engagement and satisfaction decline while burnout accelerates ^41^. The issue is often not volume alone, but low-value work that crowds out learning, recovery, and patient connection.

In medical education, entrustment and assessment systems often feel performative, with feedback reduced to paperwork and checkboxes ^42, 43^. Fragmented IT systems and redundant forms overload cognition and signal mistrust, while poorly designed physical spaces—overcrowded nurse stations, inadequate work surfaces, harsh lighting, and noise—compound fatigue and interfere with focus. Learners documenting while standing in hallways or competing for computers internalize a message—that their time and attention are expendable. These are predictable consequences of system design, not individual resilience gaps.

Programs can consolidate digital platforms, eliminate duplication, and simplify EPA workflows ^44^. Reliable, private workstations and quiet documentation zones, equipped with adequate seating, technology, and lighting, enhance both cognitive efficiency and psychological safety. Even modest design changes, like reducing hallway documentation or optimizing workspace layout, restore a sense of competence. Providing protected time and on-demand administrative support shifts culture from compliance to contribution. The aim is to eliminate duplication, simplify workflows, and design workspaces that signal respect, freeing attention for patients, feedback, and recovery.

### Tip 6. Make justice and safety integral to institutional design

Sexual harassment, abuse, and discrimination remain alarmingly prevalent in medicine, with systematic reviews reporting rates as high as 60%, particularly among women and marginalized groups ^45^. Yet institutional responses often compound harm—processes are opaque, overly procedural, and more attuned to risk management than to healing. Reports are frequently miscategorized or reframed, with greater attention paid to institutional liability than to the dignity and safety of those affected ^46^. A learner who reports a supervisor’s inappropriate behaviour may see the case downgraded to “incivility,” delaying protection and deepening disillusionment. What appears administrative is often experienced as silencing, compounding harm rather than resolving it. These failures are not separate from wellness: they predictably erode trust, frustrate psychological needs, and create moral injury, especially when people anticipate futility or reprisal.

A key indicator is whether reporting pathways restore voice and control or remove it. Embedding justice directly into institutional design—through transparent processes, trauma-informed support, and meaningful choice—restores autonomy, trust, and moral legitimacy ^47^. This includes clear timelines, trained advocates, closed loop communication to the reporter, and pattern tracking across units rather than isolating cases. When reporting pathways are transparent, supported, and closed-loop, justice functions as a trust-building resource rather than a source of further harm.

## Amplifying resources: supporting the satisfaction of basic psychological needs

Medicine is inherently rich with opportunities for meaning, growth, and connection. But even when major barriers are removed, flourishing does not arise automatically. Sustained well-being requires the deliberate cultivation of resources that replenish energy, reinforce purpose, and enable people to meet challenge with vitality rather than depletion. Enhancing wellness therefore means not only reducing harm, but actively designing environments that support autonomy, competence, and relatedness as renewable sources of motivation. The following strategies shift the focus from repair to cultivation, strengthening the conditions that allow demanding work to remain meaningful, energizing, and sustainable over time.

### Tip 7. Design workloads that replenish energy and reinforce purpose

When designed intentionally, workload becomes a vehicle for learning and identity formation rather than a source of depletion. Within JD-R, challenge demands—steep learning curves, clinical responsibility, and complex patient care—can be deeply motivating when balanced by adequate resources such as attentive supervision, timely feedback, and protected recovery time. What distinguishes growth from strain is not simply hours worked, but whether effort unfolds within coherent, educationally balanced systems ^48, 49^. In SDT terms, challenge is most sustainable when it feels supported and meaningful rather than imposed and isolating.

Resources that sustain energy include protected rest, manageable cognitive load, and opportunities for purposeful contribution. Even small design choices—quiet call rooms, access to food, and spaces conducive to documentation and decompression—shape focus and recovery. Completing meaningful clinical work with continuity (e.g., following a patient from assessment through plan) or receiving authentic appreciation reinforces competence and relatedness, re-anchoring effort to purpose. Conversely, excessive cross-coverage, redundant paperwork, and unacknowledged labour turn challenge into hindrance, draining motivation. The practical goal is not “less work,” but work that is intentionally designed so challenge remains developmental rather than depleting.

Programs can promote sustainable excellence by reducing low-value friction, distributing service demands equitably, and making meaning more visible through coaching, reflection, and shared narratives, so learners’ effort translates into growth, contribution, and recovery over time.

### Tip 8. Balance clinical responsibility with developmental support

Authentic clinical responsibility is a potent developmental resource that nourishes autonomy, competence, and relatedness. Meaningful patient care—not peripheral tasks or busy work—allows learners to experience ownership, mastery, and contribution. Within the JD-R framework, such responsibilities function as challenge demands that strengthen motivation when paired with adequate support ^50^.

The value of this balance is evident in both theory and practice. Excessive oversight can undermine autonomy and strain relationships, while removing responsibility altogether deprives learners of purpose and growth ^51^. The central task is therefore calibration: matching clinical responsibility to learners’ developing competence while ensuring reliable supervision and psychological safety. This balance allows challenge to be experienced as supportively held rather than as exposure.

Evidence supports this approach. Sawatsky *et al*. ^52^ demonstrated that entrustment aligned with competence strengthens engagement and professional identity, enabling learners to internalize responsibility as growth instead of burden. A resident trusted to lead a patient handover under guided supervision experiences responsibility as empowerment, not threat. Programs can operationalize this by scaffolding responsibility with clear expectations, timely feedback, and graduated entrustment—ensuring that responsibility functions as a renewable resource that fuels learning, identity formation, and sustained motivation.

### Tip 9. Elevate learner voice through authentic partnership

Learner leadership roles have the potential to function as powerful motivational resources—supporting autonomy, competence, and relatedness—when they provide genuine influence over decisions that shape training. When participation is symbolic rather than substantive, however, these same roles can feel hollow, offering visibility without impact and limiting their developmental value. Reviews of learner engagement in health professions education consistently note that decision-making involvement is often poorly defined and constrained, reducing its capacity to support meaningful agency ^53, 54^.

Authentic partnership transforms participation into a resource rather than a burden. Evidence from student-faculty co-design initiatives demonstrates that when learners share real responsibility and influence, engagement, trust, and collective resilience increase ^55^. These roles allow learners to develop leadership skills, systems thinking, and professional identity while contributing to institutional improvement. In this way, partnership supports not only individual growth but also organizational learning.

Programs can maximize the value of learner voice by designing leadership roles with clear scope, continuity, and authority. Effective partnerships have structures that persist beyond individual cohorts, processes in which learner input precedes key decisions, and alignment between institutional priorities and learners’ developmental goals. Learners are already leading innovation in areas like equity, digital education, and sustainability, yet these contributions often remain fragmented or underutilized ^56^. When institutions share decision ownership and make impact visible, learner partnership becomes a renewable resource—fueling motivation, belonging, and shared accountability rather than functioning as unpaid cognitive or emotional labour ^57^.

### Tip 10. Deliver feedback that builds competence and agency

In many programs, feedback is experienced as inconsistent, bureaucratic, or primarily evaluative rather than developmental. When delivered sporadically or framed as judgment, feedback functions as a hindrance demand: it heightens anxiety, undermines confidence, and contributes to disengagement rather than learning. Educators may also struggle with delivering corrective or “negative” feedback, fearing it will feel confrontational or demoralizing, often leading to avoidance or dilution that further reduces its usefulness. Such feedback practices are often perceived as performance threats rather than supportive resources.

An alternative approach is to reconceptualize feedback as a resource for competence and agency. High frequency, low-stakes feedback—embedded in bedside observation, coaching conversations, or reflective portfolios—helps learners calibrate performance and build mastery over time. Programs that integrate brief goal-setting and reflection consistently report higher engagement and more accurate self-assessment than those relying on episodic input ^58^. Within SDT, even corrective feedback can enhance motivation when it is delivered with empathy, clarity, and clear rationale ^59^. When framed as guidance rather than judgment, feedback transforms anxiety into trust, supporting internalization and sustained motivation ^60^. In JD-R terms, feedback shifts from a hindrance demand to a protective resource that promotes confidence, learning, and resilience.

Operationalizing this shift requires intentional practices at both the learner and faculty levels. A practical antidote to evaluative feedback cultures is the normalization of agentic engagement: learners actively shape feedback by clarifying goals, inviting critique, and contributing ideas for improvement ^61– 63^. When faculty respond with curiosity rather than verdict—for example, by asking, “What would be most useful for you to hear right now?”—feedback becomes collaborative rather than surveillant, supporting competence and autonomy ^64, 65^. Programs can reinforce this approach by explicitly teaching learners how to request feedback effectively and training faculty to respond in ways that avoid slipping into performance policing. The aim is not simply *more* feedback, but feedback that is usable, respectful, and oriented toward growth.

### Tip 11. Foster belonging through inclusion and psychological safety

Training environments must help learners feel safe, seen, and valued if motivation and learning are to be sustained. Belonging is shaped not only through interpersonal interactions but also through physical and symbolic cues, such as inclusive imagery, accessible design, and welcoming communal spaces, which can communicate respect and legitimacy as powerfully as formal policies. When learners are able to bring their full selves to training, relatedness deepens, competence grows, and motivation strengthens. Within the JD-R framework, inclusion and psychological safety operate as core job resources that buffer stress and sustain engagement. In SDT terms, belonging is not a “nice to have,” but a foundational condition for motivated learning in high-stakes environments ^66, 67^.

Empirical evidence consistently supports this framing. Ganotice *et al*. ^68^ demonstrated that curricula explicitly supporting autonomy, competence, and relatedness improved teamwork, engagement, and collective goal attainment. McClintock *et al*. ^69^ showed that autonomy-supportive teaching was critical to psychological safety, whereas disinterest or dismissiveness rapidly fractured it. Notably, once psychological safety was lost, learners rarely reported it being fully restored, highlighting the importance of proactive, rather than reactive, climate building. Small everyday behaviours matter: faculty acknowledging uncertainty, inviting questions, or sharing their own learning missteps, can rebuild trust in ways that policies alone cannot. Leaders play a key role by protecting time for teaching, debriefing, and follow-up, rather than treating relational work as optional or expendable.

Programs can further embed inclusion by pairing anti-bias education with structural supports, such as amplifying underrepresented voices and creating meaningful leadership pathways for marginalized groups. Faculty development should emphasize empathy, humility, and reflective practice; without these, diversity initiatives risk remaining aspirational rather than embodied. Psychological safety is most durable when it is woven into routine practices—how rounds are conducted, how questions are received, and how mistakes are discussed—rather than invoked as a slogan. Leaders can reinforce this by evaluating and supporting teaching climates, not only individual performance, signaling that belonging and safety are shared responsibilities and institutional priorities ^67^.

### Tip 12. Build relational continuity through faculty-learner partnerships

Sustained, trusting relationships between faculty and learners are foundational to motivation and well-being. Longitudinal integrated clerkships (LICs), in which learners remain with the same team or preceptor over time, exemplify this continuity and are associated with stronger feedback, coaching, and mentorship ^70^. A BEME systematic review found consistent benefits for learning, professional development, and satisfaction ^71^. When full LICs are not feasible, similar advantages can be achieved through stable team assignments, consistent preceptors, or schedules that preserve relational continuity. Within the JD-R framework, such continuity functions as a high-yield job resource, reducing uncertainty while increasing the accessibility and effectiveness of support.

Relational continuity enables supervision to evolve from episodic oversight into a developmental partnership. Over time, faculty gain a more accurate understanding of learners’ abilities and trajectories, allowing for fairer assessment, more credible feedback, and support that is both timely and personalized ^58^. Repeated interaction with consistent role models also supports professional identity formation, as learners internalize values, norms, and ways of being in practice ^72^. From an SDT perspective, these relationships foster psychological safety and need satisfaction, enabling learners to stretch beyond comfort zones with confidence and trust. Faculty who follow learners longitudinally can more authentically recognize growth, set expectations, and model vulnerability, demonstrating that expertise and humility can coexist.

Faculty workload concerns are legitimate, but continuity can be mutually sustaining ^73^. Faculty mentors often report greater meaning and connection when they are able to witness learners’ progression over time. Institutions can support these partnerships through deliberate pairing strategies, protected mentorship time, and faculty development focused on coaching and longitudinal assessment. Investing in relational continuity helps ensure that supervision is not merely evaluative, but a shared developmental enterprise that strengthens learning, fairness, and long-term engagement.

## Conclusion: From theory to transformative practice

Medical education has made meaningful progress in addressing physician burnout, yet efforts often remain fragmented and overly focused on individual coping. Across the literature, countless interventions show promise but remain difficult to replicate and theoretically underpowered. Self-Determination Theory (SDT) and the Job Demands-Resources (JD-R) model can help unite these disparate efforts under a single explanatory framework, offering shared language, validated metrics, and a scalable roadmap for reform. Their integration enables educators and institutions to coordinate strategies, compare outcomes, and build cumulative knowledge grounded in motivation science rather than intuition.

By contrast, SDT and JD-R provide a coherent foundation for sustainable reform, clarifying not only why motivation matters, but how to cultivate it across systems. This paper outlined twelve interconnected strategies that reduce hindrance demands and strengthen resources, moving wellness efforts beyond surface-level fixes toward enduring cultural transformation. The goal is not to remove challenge, but to ensure that high standards and hard work take place within climates that support autonomy, competence, and relatedness.

Ultimately, the call to align systems with human needs is not only a matter of design—it is a matter of conscience. Physicians and learners cannot flourish in systems that drain their sense of purpose or isolate them from genuine connection. Supporting motivation is therefore a professional, ethical, and human imperative—a reminder that well-being and excellence arise from the same moral foundation. When institutions honour this truth—through the spaces they create, the policies they craft, and the relationships they sustain—medical education can move beyond endurance toward a culture of vitality, integrity, and compassion.

## Ethics and consent

Ethical approval and consent were not required.

## Data Availability

No data are associated with this article.
