# Supplementary material for: Flourishing by Design: Applying Self-Determination Theory and the Job Demands-Resources Model to Systems-Level Wellness in Medical Education
Source: MedEdPublish (2016). 2026 Jan 20;15:18. Originally published 2025 Apr 16. [Version 3] doi: 10.12688/mep.20886.3 (PMC12616115; doi:10.12688/mep.20886.3)
Supplement: Peer review history

# Reviewer report: Reviewer response for version 3

**DOI:** 10.21956/mep.23004.r45210

**Published:** 2026-01-21

**Authors:** Binata Mukherjee

**Affiliations:** 1 University of South Alabama, Mobile, Alabama, USA

No further comments

If evidence from practice is presented, are all the underlying source data available to ensure full reproducibility?

Not applicable

Is the topic of the practical tips discussed accurately in the context of the current literature

Partly

Are all factual statements correct and adequately supported by citations?

Partly

Are the conclusions drawn balanced and justified on the basis of the presented arguments?

Partly

Are arguments sufficiently supported by evidence from the published literature and/or the authors’ practice?

Partly

Reviewer Expertise:

Provider burnout, Provider wellness, Leadership education and training, Coaching

I confirm that I have read this submission and believe that I have an appropriate level of expertise to confirm that it is of an acceptable scientific standard.

# Reviewer report: Reviewer response for version 2

**DOI:** 10.21956/mep.22907.r44206

**Published:** 2025-12-11

**Authors:** Binata Mukherjee

**Affiliations:** 1 University of South Alabama, Mobile, Alabama, USA

Thank you for addressing all the concerns I had raised. This manuscript is well organized, clear, and easy to follow.

If evidence from practice is presented, are all the underlying source data available to ensure full reproducibility?

Not applicable

Is the topic of the practical tips discussed accurately in the context of the current literature

Partly

Are all factual statements correct and adequately supported by citations?

Partly

Are the conclusions drawn balanced and justified on the basis of the presented arguments?

Partly

Are arguments sufficiently supported by evidence from the published literature and/or the authors’ practice?

Partly

Reviewer Expertise:

Provider burnout, Provider wellness, Leadership education and training, Coaching

I confirm that I have read this submission and believe that I have an appropriate level of expertise to confirm that it is of an acceptable scientific standard.

# Reviewer report: Reviewer response for version 2

**DOI:** 10.21956/mep.22907.r44205

**Published:** 2025-11-13

**Authors:** Shaun Prentice

**Affiliations:** 1 GP Training Research Team, Royal Australian College of General Practitioners, East Melbourne, Victoria, Australia; 2 The University of Adelaide, Adelaide, South Australia, Australia

Dear Dr Neufeld,

 Thank you for incorporating my suggested edits into your manuscript, I believe they have substantially strengthened this already excellent paper.  Congratulations on producing this piece.

If evidence from practice is presented, are all the underlying source data available to ensure full reproducibility?

Not applicable

Is the topic of the practical tips discussed accurately in the context of the current literature

Yes

Are all factual statements correct and adequately supported by citations?

Yes

Are the conclusions drawn balanced and justified on the basis of the presented arguments?

Yes

Are arguments sufficiently supported by evidence from the published literature and/or the authors’ practice?

Partly

Reviewer Expertise:

Medical education, medical trainee burnout and wellbeing, clinical psychology

I confirm that I have read this submission and believe that I have an appropriate level of expertise to confirm that it is of an acceptable scientific standard.

# Reviewer report: Reviewer response for version 1

**DOI:** 10.21956/mep.22365.r43343

**Published:** 2025-09-25

**Authors:** Binata Mukherjee

**Affiliations:** 1 University of South Alabama, Mobile, Alabama, USA

Burnout and wellness are topics of concern for physicians and trainees, and therefore deserve due attention. It is commendable that the author is attempting to provide theory-informed practical tips. My suggestions for revisions are essentially meant to strengthen the manuscript.

 1. Burnout and wellness have been used interchangeably based on the underlying assumption that addressing/reducing burnout leads to wellness, which may not necessarily be true. The two concepts are distinct. Absence of one does not mean presence of the other. I would expect this assumption to be made explicit upfront. 

 2. The paper is mostly focused on graduate medical education and not the entire continuum of medical education of UME and GME. It would be great to make that clear upfront so that readers are not kept guessing. This may mean rewriting sentences that may have hinted at UME, e.g., Tip 11. Also in some places the entire physician population have been included which may be redirected to physician trainees; e.g., suicide rate etc may be given for trainees specifically.  

 3. While most of the paper is based on theory-driven concepts, only in the conclusion 'evidence-based' is introduced in association with theory-driven. As the previous reviewers have pointed out, it is desirable to clearly delineate and point out which ones are evidence-based vs which ones are theory-driven claims.   

 4. Tip 3 and Tip 4 - both refer to hierarchy and may be either combined or made more distinct.

 5. While some tips clearly address hindrances, some increase resources, some do both. For example, Tip 8 and Tip 1, Tip 9 and Tip 2, Tip 11 and Tip 3 do both. So the paper may reorganized into three groups: hindrances, resources and both. 

 5. While it is desirable to reduce administrative duties during training, in reality these are inevitable aspects of one's job after graduation especially if one chooses to be in academic medicine. Even though it is not the direct scope of the paper, it might be relevant to include some thoughts on the downstream effects if these reforms were to take place. 

 To conclude, this is a well written manuscript that should help medical educators in GME.

If evidence from practice is presented, are all the underlying source data available to ensure full reproducibility?

Not applicable

Is the topic of the practical tips discussed accurately in the context of the current literature

Partly

Are all factual statements correct and adequately supported by citations?

Partly

Are the conclusions drawn balanced and justified on the basis of the presented arguments?

Partly

Are arguments sufficiently supported by evidence from the published literature and/or the authors’ practice?

Partly

Reviewer Expertise:

Provider burnout, Provider wellness, Leadership education and training, Coaching

I confirm that I have read this submission and believe that I have an appropriate level of expertise to confirm that it is of an acceptable scientific standard, however I have significant reservations, as outlined above.

# Response

**Published:** 2025-10-27

**Authors:** Adam Neufeld

**Affiliations:** University of Calgary, Canada

I would like to express my sincere gratitude to the reviewer for their thoughtful and constructive feedback. I appreciate their recognition of the manuscript’s theoretical grounding, practical relevance, and potential value to graduate medical education. Each of the reviewer’s comments has been carefully considered and used to strengthen the conceptual clarity, structure, and applicability of the paper. Below, I outline the specific revisions made in response.

1. **Clarifying the distinction between burnout and wellness**
  I fully agreed with the reviewer’s important point that burnout and wellness are distinct constructs and should not be used interchangeably. In the revised manuscript, this distinction is now made explicit in both the **Abstract** and **Introduction**. I clarified that the reduction of burnout does not automatically confer wellness, and that thriving represents a qualitatively distinct state characterized by vitality, meaning, and engagement. This conceptual separation is now framed as central to the paper’s argument—that the goal of reform is not merely to reduce distress but to actively cultivate flourishing.
2. **Clarifying scope and continuum of focus (UME vs. GME)**
  I have clarified early in the **Introduction** that the paper’s primary focus is on graduate medical education (GME), while acknowledging that many of the principles and strategies are applicable across the continuum of medical training. This revision prevents ambiguity regarding scope and audience. In addition, references and examples that previously implied a broader physician population have been adjusted to focus specifically on trainees where appropriate. For example, mentions of burnout and suicide rates now specify findings from resident or trainee populations.
3. **Distinguishing theory-driven and evidence-based claims**
  Consistent with this reviewer’s and others’ feedback, the manuscript now explicitly distinguishes between **empirically supported** and **theoretically informed** recommendations. Within each tip, I identify whether statements derive primarily from SDT and JD-R theory or are backed by empirical evidence such as large-scale studies, systematic reviews, or applied interventions. This enhances transparency and helps readers understand the empirical grounding of the recommendations.
4. **Clarifying the distinction between Tip 3 and Tip 4**
  The reviewer correctly noted overlap between **Tip 3 (Flatten Hierarchies and Diminish Competitive Climates)** and **Tip 4 (Replace Controlling Faculty Behaviours with Autonomy Support)**. In the revised manuscript, these tips have been further differentiated. Tip 3 now focuses on *structural and cultural hierarchies* that shape group dynamics and climate, while Tip 4 emphasizes *interpersonal behaviours and leadership styles* that influence motivation and psychological need support at the faculty–learner level. The refined framing makes their conceptual boundaries clearer and preserves their complementary contributions.
5. **Clarifying rationale for organizational structure of tips**
  The reviewer suggested reorganizing the tips into three groups (hindrances, resources, and both). I considered this carefully and have retained the original two-section organization—six tips addressing hindrance demands and six amplifying resources—because this structure aligns directly with the Job Demands–Resources model and is consistent with frameworks used in the organizational psychology literature. However, I revised several transition statements to acknowledge that some strategies (e.g., Tips 1, 8, 9, and 11) function across both domains by simultaneously reducing barriers and enhancing supports. This clarification preserves theoretical coherence while recognizing the multidimensional nature of certain reforms.
6. **Addressing downstream implications of reduced administrative burden**
  I appreciated the reviewer’s thoughtful suggestion to reflect on the downstream implications of reducing administrative duties, especially for those who pursue academic medicine. In **Tip 5 (Streamline Bureaucracy to Reduce Cognitive and Emotional Load)**, I have added a brief discussion acknowledging that while administrative tasks are inevitable in professional practice, their design, relevance, and proportionality during training shape long-term attitudes toward such work. The revised text emphasizes that the goal is not to eliminate administrative responsibility but to align it with purpose, efficiency, and developmental value—preparing trainees for sustainable practice rather than shielding them from reality.

Once again, I thank the reviewer for their constructive insights and collegial tone. Their feedback has strengthened the conceptual precision, internal organization, and practical utility of the manuscript, making it a clearer and more valuable resource for educators and leaders in graduate medical education.

# Reviewer report: Reviewer response for version 1

**DOI:** 10.21956/mep.22365.r43340

**Published:** 2025-09-15

**Authors:** Luca Pirrotta

**Affiliations:** 1 Institute of Management and Department EMbeDS, Scuola Superiore Sant'Anna, Pisa, Italy

Dear Editor,

 Thank you for the opportunity to review this manuscript.

 I greatly appreciate authors ‘effort in addressing the critical issue of physician wellness in medical education. The integration of Self-Determination Theory (SDT) and the Job Demands–Resources (JD-R) model into a set of actionable recommendations is both timely and highly relevant. The manuscript is well-structured and has the potential to become a significant reference in the literature.

 Overall, I believe this work is of publishable quality. At the same time, I would encourage a few revisions that could further enhance its clarity, accessibility, and empirical grounding:

 At times, it is not entirely clear which recommendations are supported by empirical evidence, and which are primarily grounded in theoretical reasoning. I recommend making this distinction more explicit (e.g., by indicating which claims are “empirically supported” vs. “theoretically informed”). This would increase the transparency and impact of your framework.

 Also, given that many readers may not have a background in psychology, a slightly more detailed and accessible introduction to the two models would be helpful. Briefly defining the key constructs (autonomy, competence, relatedness; job demands vs. job resources) and illustrating them with examples from medical education would make the article more approachable to faculty and administrators.

 Some tips are presented with rich, concrete examples, while others are more general. Adding a few more practical illustrations where the recommendations are brief would improve balance and ensure that all tips are equally actionable.

 Also, while most tips apply broadly, Tip 10 (on inclusion and belonging) seems oriented mainly to undergraduate medical education. I encourage you to broaden this recommendation to also include residency settings. Similarly, Tip 11 could highlight how faculty–learner partnerships extend beyond medical school. This would strengthen the applicability of your framework across the entire continuum of training.

 Finally, your manuscript rightly highlights the importance of reducing excessive workload and administrative burden. This point could be further reinforced by engaging with empirical studies on staffing adequacy within the JD-R framework. For example, recent large-scale evidence from public healthcare organizations shows how both perceived and objective staffing needs directly affect workload sustainability and job satisfaction, and how resources such as leadership, training and adequate equipment can buffer these effects (https://doi.org/10.1108/MD-07-2024-1718). Incorporating insights from this type of research would provide additional empirical grounding to your recommendations (especially Tips 5 and 6) and underline the systemic importance of HRM practices alongside educational reforms.

 A few sentences could be simplified to avoid ambiguity, as already stated by previous reviewer.

 To conclude, this is a highly valuable and well-written manuscript. The above revisions are intended to strengthen its clarity, accessibility, and empirical support, thereby maximizing its contribution to the field. I strongly support publication once these points have been addressed.

 Sincerely,

 Luca Pirrotta

If evidence from practice is presented, are all the underlying source data available to ensure full reproducibility?

Not applicable

Is the topic of the practical tips discussed accurately in the context of the current literature

Yes

Are all factual statements correct and adequately supported by citations?

Yes

Are the conclusions drawn balanced and justified on the basis of the presented arguments?

Partly

Are arguments sufficiently supported by evidence from the published literature and/or the authors’ practice?

Partly

Reviewer Expertise:

Healthcare Management, HRM, Employees' Wellbeing, Organizational Behaviour

I confirm that I have read this submission and believe that I have an appropriate level of expertise to confirm that it is of an acceptable scientific standard, however I have significant reservations, as outlined above.

## References

[1] : Exploring the role of staffing needs in JD-R theory: evidence from public healthcare organizations. *Management Decision* .2025;63(13) : 10.1108/MD-07-2024-1718 282-301 10.1108/MD-07-2024-1718

# Response

**Published:** 2025-10-27

**Authors:** Adam Neufeld

**Affiliations:** University of Calgary, Canada

I am deeply grateful to the reviewer for their thoughtful and generous evaluation of the manuscript. I greatly appreciate the recognition of its structure, clarity of purpose, and potential contribution to the field. The reviewer’s constructive feedback has been invaluable in refining the paper’s accessibility, empirical grounding, and practical impact. Below, I outline how each of the suggested revisions has been incorporated.

1. **Clarifying empirical vs. theoretical foundations**
  I fully agreed with the reviewer’s observation that readers would benefit from a clearer distinction between empirically supported recommendations and those primarily grounded in theory. The revised manuscript now explicitly differentiates these throughout the text. Within the twelve tips and the theory sections, I have clarified when claims are *empirically supported* (e.g., through multi-site studies, systematic reviews, or quantitative evidence) versus *theoretically informed* (e.g., directly derived from SDT or JD-R principles). This change enhances transparency and allows readers to better appreciate the strength and source of each recommendation.
2. **Enhancing accessibility and defining key constructs**
  As recommended, I have expanded and simplified the introductory explanations of both **Self-Determination Theory (SDT)** and the **Job Demands–Resources (JD-R)** model. These sections now include clearer definitions of autonomy, competence, and relatedness, as well as the distinctions between job demands and job resources, challenge versus hindrance demands, and need satisfaction versus need frustration. Each construct is illustrated with concrete examples from medical education, making the framework more accessible for readers without a psychology background—especially faculty and administrators.
3. **Balancing theoretical and practical depth across tips**
  I have reviewed all twelve tips to ensure that each includes both theoretical grounding and concrete, actionable examples. In particular, Tips 4, 7, and 9 were enriched with specific, contextually relevant illustrations drawn from applied practice and empirical literature. This creates a more even distribution of depth and makes each tip equally actionable for educators and institutional leaders.
4. **Broadening applicability across the continuum of training**
  In response to the reviewer’s excellent suggestion, I have revised **Tip 10** (“Deliver Feedback That Builds Competence and Agency”) and **Tip 11** (“Foster Belonging Through Inclusion and Psychological Safety”) to ensure they are explicitly applicable to postgraduate (residency) as well as undergraduate training. Each now includes references and examples relevant to residency environments, reinforcing the framework’s applicability across the full continuum of medical education.
5. **Integrating empirical evidence on staffing and workload**
  I appreciate the reviewer’s insightful recommendation to strengthen the empirical basis of **Tips 5 and 6** by engaging with research on staffing adequacy and workload sustainability. These sections now explicitly reference recent large-scale studies examining how staffing resources, leadership, and infrastructure buffer the impact of excessive workload and administrative burden within the JD-R framework. I have also integrated the reviewer’s cited work (Pirrotta et al., 2024) to highlight the systemic importance of human resource management and organizational design alongside educational reform.
6. **Improving clarity and readability**
  Consistent with the feedback from both reviewers, I undertook a careful stylistic review to simplify and clarify complex sentences throughout the manuscript. Ambiguous phrasing has been refined to ensure that key concepts are accessible and unambiguous on first read. These revisions improve flow and comprehension while preserving scholarly precision.

Once again, I sincerely thank the reviewer for their detailed and constructive feedback. Their comments have greatly enhanced the clarity, accessibility, and scholarly rigor of the manuscript, and I am confident that the revisions have strengthened its contribution to advancing systems-level wellness reform in medical education.

# Reviewer report: Reviewer response for version 1

**DOI:** 10.21956/mep.22365.r41583

**Published:** 2025-05-13

**Authors:** Shaun Prentice

**Affiliations:** 1 GP Training Research Team, Royal Australian College of General Practitioners, East Melbourne, Victoria, Australia; 2 The University of Adelaide, Adelaide, South Australia, Australia

Thank you for the opportunity to review this paper.  I am thrilled to see the application of JDR and SDT in the one paper to systemic issues facing medical trainee wellbeing and burnout; this represents a very significant contribution to the literature and I think this could easily become a seminal paper.  I would like to thank the author for writing on such an important topic.

 I think the structure of the article is excellent; the use of JDR to structure the tips and then weaving SDT through the tips themselves makes for a very approachable read.  I am also very pleased to see the inclusion not only of frustration of BPN but also how to enhance BPN; this dual focus on ill- and well-being is fantastic and just what the literature needs.

 One area that could be improved is linkages to literature.  It isn't always clear what aspect of a claim the citations are supporting.  I understand this will increase the wordiness of the piece, but I think it's important that the evidence is more clearly described so the reader understands how recommendations are evidence-based vs theory-informed.

 I have a good working knowledge of both SDT and JDR, but I think it would be worthwhile to provide some more detail about both of these models.  I think this is necessary given the intended audience would presumably be medical faculty and program administrators, who may not have the same background in this psychological literature.

 There are a few phrases that are difficult to interpret on first read, for example:

 1. "medical education must prioritize reforms that reduce job demands that frustrate basic psychological needs" - the double 'that' in this sentence requires a bit of mental gymnastics to interpret

 2. "Medical curricula often impose rigid, one-size-fits-all frameworks that limit learners' autonomy, which is essential for intrinsic motivation and well-being" - the 'which' here makes it sound on first read that a rigid framework is essential for intrinsic motivation

 I'd recommend the phrasing using in the article to make these points clearer, perhaps some wording changes or dividing ideas into multiple sentences to maximise clarity.  The ideas being presented are excellent, but they are let down by the wordsmithing.

 Regarding tip 10, up until now I've seen how all the tips could be applied throughout medical education (i.e., including residency), but tip 10 exclusively talks about medical school.  I would encourage the author to consider whether tip 10 could also be applied to residency settings (which I think it could) and provide some guidance in this respect.  This would help maximise the paper's applicability to the full continuum of medical education.  This is also the case, but to a lesser extent, for tip 11.

If evidence from practice is presented, are all the underlying source data available to ensure full reproducibility?

Not applicable

Is the topic of the practical tips discussed accurately in the context of the current literature

Yes

Are all factual statements correct and adequately supported by citations?

Yes

Are the conclusions drawn balanced and justified on the basis of the presented arguments?

Yes

Are arguments sufficiently supported by evidence from the published literature and/or the authors’ practice?

Partly

Reviewer Expertise:

Medical education, medical trainee burnout and wellbeing, clinical psychology

I confirm that I have read this submission and believe that I have an appropriate level of expertise to confirm that it is of an acceptable scientific standard, however I have significant reservations, as outlined above.

# Response

**Published:** 2025-06-25

**Authors:** Adam Neufeld

**Affiliations:** University of Calgary, Canada

Dear Dr. Prentice, Thank you very much for your thoughtful and constructive feedback. I greatly appreciate your support for the paper and your recognition of its potential contribution to the literature on systemic wellness reform in medical education. As I prepare the manuscript for its second review, I plan to implement several revisions that address your suggestions:

1. **Enhance clarity of theoretical concepts:** I will add concise, accessible overviews of Self-Determination Theory and the Job Demands-Resources model so that readers—regardless of their psychology background—can fully engage with the ideas presented.
2. **Improve citation transparency:** I will clarify the relationship between citations and claims throughout the paper, noting how each reference supports the point being made and distinguishing theory-informed recommendations from those with direct empirical backing.
3. **Refine language for clarity:** Ambiguous or complex phrases will be revised for smoother readability and clearer interpretation, particularly where prior wording may have obscured intent or meaning.
4. **Broaden applicability to residency:** All tips, 10 and 11 in particular, will be expanded to show how strategies apply to residency training, ensuring relevance across the continuum of medical education.

I believe these planned changes will strengthen the manuscript’s clarity, accessibility, and practical value for medical educators and institutional leaders. Thank you again for your generous engagement and for helping to shape this work. Sincerely, Adam Neufeld

# Response

**Published:** 2025-10-27

**Authors:** Adam Neufeld

**Affiliations:** University of Calgary, Canada

I would like to sincerely thank the reviewer for their thoughtful and encouraging feedback. I am deeply grateful for the generous appraisal of the paper’s structure, theoretical framing, and contribution to the literature. The reviewer’s comments have strengthened the manuscript considerably. Below, I summarize how each of the key points raised has been addressed in the revised version.

1. **Clarifying evidence and theory linkages**
  I fully agreed with the reviewer’s observation that it was not always clear which claims were evidence-based versus theory-informed. In the revised manuscript, I have explicitly identified the nature of each assertion, clarifying where recommendations draw from empirical findings (e.g., large-scale, multi-site, or systematic reviews) and where they are grounded in SDT or JD-R theory. I have expanded several evidence summaries within the tips to explicitly state the study context and outcomes (e.g., in Tips 1–6 and 8–10), and revised the introduction and theory sections to orient readers to how these frameworks inform practical recommendations.
2. **Expanding explanation of SDT and JD-R for non-specialist readers**
  Recognizing that many readers may not have prior familiarity with these frameworks, I have enhanced both the **SDT** and **JD-R** sections to provide more accessible, applied explanations. These now include clearer definitions of key constructs (e.g., basic psychological needs, challenge vs. hindrance demands, dual-process pathways), practical examples from medical education, and brief summaries of supporting empirical evidence. The revisions ensure that faculty, administrators, and educators new to motivational theory can readily grasp how each framework functions and why their integration is valuable.
3. **Improving clarity and readability of complex sentences**
  I carefully reviewed the manuscript to address the reviewer’s helpful examples of ambiguous phrasing. Sentences such as *“medical education must prioritize reforms that reduce job demands that frustrate basic psychological needs”* and *“Medical curricula often impose rigid, one-size-fits-all frameworks that limit learners' autonomy, which is essential for intrinsic motivation and well-being”* were rewritten for clarity and smoother syntax. Throughout the paper, multi-clause sentences were simplified or divided to improve flow and readability. These edits collectively make the paper more accessible without compromising scholarly precision.
4. **Ensuring applicability across the continuum of medical education**
  I have revised **Tip 10** (“Deliver Feedback That Builds Competence and Agency”) to explicitly include examples and language relevant to residency and postgraduate training, emphasizing its applicability beyond undergraduate settings. **Tip 11** (“Foster Belonging Through Inclusion and Psychological Safety”) was similarly adjusted to broaden its scope across the continuum. These refinements align with the reviewer’s valuable suggestion to enhance the paper’s relevance for the entire spectrum of medical education.
5. **General stylistic refinements and consistency**
  Beyond these major revisions, I also undertook a comprehensive line edit to improve consistency of tone, flow, and theoretical integration throughout. The updated manuscript maintains parallel structure across the twelve tips and ensures alignment between the introduction, theory sections, and conclusion.

Once again, I wish to thank the reviewer for their generous recognition of the paper’s potential and for their constructive recommendations, which have greatly improved its clarity, rigour, and impact.
